# A multi-criteria GIS model for suitability analysis of locations of decentralized wastewater treatment units: case study in Sulaimania, Iraq

**DOI:** 10.1016/j.heliyon.2019.e01355

**Published:** 2019-03-18

**Authors:** Ako Rashed Hama, Rafea Hashim Al-Suhili, Zeren Jamal Ghafour

**Affiliations:** a Civil Eng. Dept., Komar University of Science & Technology, Sulaimania, Iraq; b Civil Eng. Dept., City College of New York, New York, USA; c Irrigation Eng. Dept., College of Engineering, University of Sulaimania, Sulaimania, Iraq

**Keywords:** Environmental science, Geography

## Abstract

Sulaimania is a City located in Kurdistan region in the north of Iraq. The city is facing a lack of water, and it will reach a very critical condition shortly. One of the potential solutions is to reuse the treated wastewater for non-direct human uses such as irrigation, washing, firefighting, groundwater recharging, and others. There is no sewage treatment plant in the city. The wastewater flows into a stream through some sewer outlets, and that causes big environmental issues. Decentralized wastewater treatment units (DTUs) are suggested to solve the issue. The treated wastewater will be used for the irrigation of the green areas of the city. The selected plant type is Extended Aeration treatment system, which is recommended for residential areas. Specifying the locations of the treatment units is very important from environmental, social and technical aspects. The main objective of this study is to select the best suitable places for the DTUs. Preliminary selections of 134 nominated areas for DTU locations were made in different places in the city. The locations are distributed into 10 groups near the main sewer pipes of the city. A model is created to evaluate those selected locations and eliminate the non-suitable locations by using GIS software integrated with the Analytical Hierarchy Process (AHP). Five criteria were used in the model, which are, (1) The size of the available lands, (2) The distance from the decentralized units to the green areas (3) Population density around the decentralized treatment unit locations, (4) The slope of the land and (5) Depth of the main sewer pipe at the nominated area. In addition, the model adopted two restriction factors, which are: (1) The distance from the decentralized treatment unit to the buildings should not be less than 10 m and (2) The distance between the main sewer pipes and the treatment units are taken to be <50 m. The results of the suitability analysis produced six classes of suitability levels of the nominated areas started from restricted to extremely suitable. The suitability percentages of the 6 classes of the total nominated areas were found to be; 8.5% (6.95 ha) restricted, 0.4 % (0.23 ha) moderately suitable, 12.8% (10.50 ha) suitable, 38.8% very suitable (31.60 ha), 32.2% (26.33 ha) highly suitable and 7.3% (5.92 ha) extremely suitable. Each nominated area has more than one suitability class. Normalized Weighted Average (NWAV) of the suitability level percentage of each nominated area is found. The values of the NWAV are ranged from 0.0 to 1.0, and the selection of final DTUs locations will be for areas that have NWAV larger than 0.5. Optimum 30 suitable locations are selected out of the 134 nominated areas.

## 1 Introduction

Lack of sufficient available water resources to cover the requirements of a city is one of the crucial problems nowadays all over the world. Decentralized wastewater treatment system is considered as a powerful solution to the problem of water shortage. The treated wastewater could be reused for many purposes such as irrigation, groundwater recharging, car washing, industrial uses, and firefighting. The locations of the treatment units are critical to getting the best benefit from the reused water. Also selecting the treatment unit's position should not create any health hazard to the community (Gutterer et al., 2009). Some essential criteria should be considered when selecting the best location such as; environmental standards, social aspects, cost, and other technical details. Moreover, land availability inside the city is another important factor that will effect on selecting the location of the treatment units. First, it is required to do a careful study and collect information to select appropriate sites for the decentralized treatment units (EPA, 1980). After collecting the required data, the right techniques should be used to find suitable locations. One of the methods is the Analytical Hierarchy Process (AHP), which is used widely in decision analysis. AHP is presented by (Saaty, 1980), based on the comparison of the importance of two elements. Combining GIS with Multi-Criteria Decision Analysis (MCDA) is a powerful method in land evaluation. Many factors are usually taken when using AHP in selecting the suitable land location (Krishnaveni and Deepa, 2012) a used GIS with AHP to build a multi-criterion model to select appropriate locations for the decentralized wastewater units in Chennai area in India. Six thematic layers were selected which are: (1) Land use (land availability), (2) Population density, (3) Soil type, (4) The land slope, (5) Cost and (6) Technology (Ansari and Ahmadi, 2017). Adopted AHP using the Expert Choice 11 software for selecting suitable locations of decentralized wastewater units in Qom city in Iran. The criteria that been selected are:(1) Population density, (2) Land slope, (3) Land use, and (4) Reusing, concerning the environmental, economic, and social conditions of Qom (Gemitzi et al., 2007). a used GIS for siting areas for stabilization pond system (SP) to be used for the treatment of wastewater of rural regions in Thrace (Northeast Greece). The factors that considered in the selection methodology are; (1) Environmental criteria, (2) Land topography, (3) Land use, (4) Geological formation, (5) Distance from the SP units to the major rivers, (6)Distance to the existing cities and villages, and (7) Effluent characteristics (Meinzinger, 2003). selected suitable locations to use Land Application Method for treated sewage produced from a wastewater treatment plant in Christchurch city in New Zealand by using GIS. The selection was based on many factors which are; (1) Social acceptability, (2) Soil type, (3) Economic, (4) Weather (5) Land slope and (6) Environmental factors. Other researchers used other methods such genetic Algorithm GA to select the suitable locations for decentralized treatment units (Naik, 2014). Developed an optimization model using Genetic Algorithm (GA) to find optimum design configurations of decentralized wastewater treatment system regarding best locations and number of treatment units to get minimum cost and highest benefit.

This work aims to find the optimal locations for the decentralized treatment units in Sulaimania City in Iraq to be reused for irrigation by using AHP combined with GIS. Specifying those locations inside a city like Sulaimania needs a careful study as most of the districts are residential and the population densities are different from region to another region. Moreover, the city land is mountainous, and there is a big difference in the land levels. Finally, the locations of the existing sewer network are also considered as one of the significant factors.

## 2 Study area

**Fig. 1:**
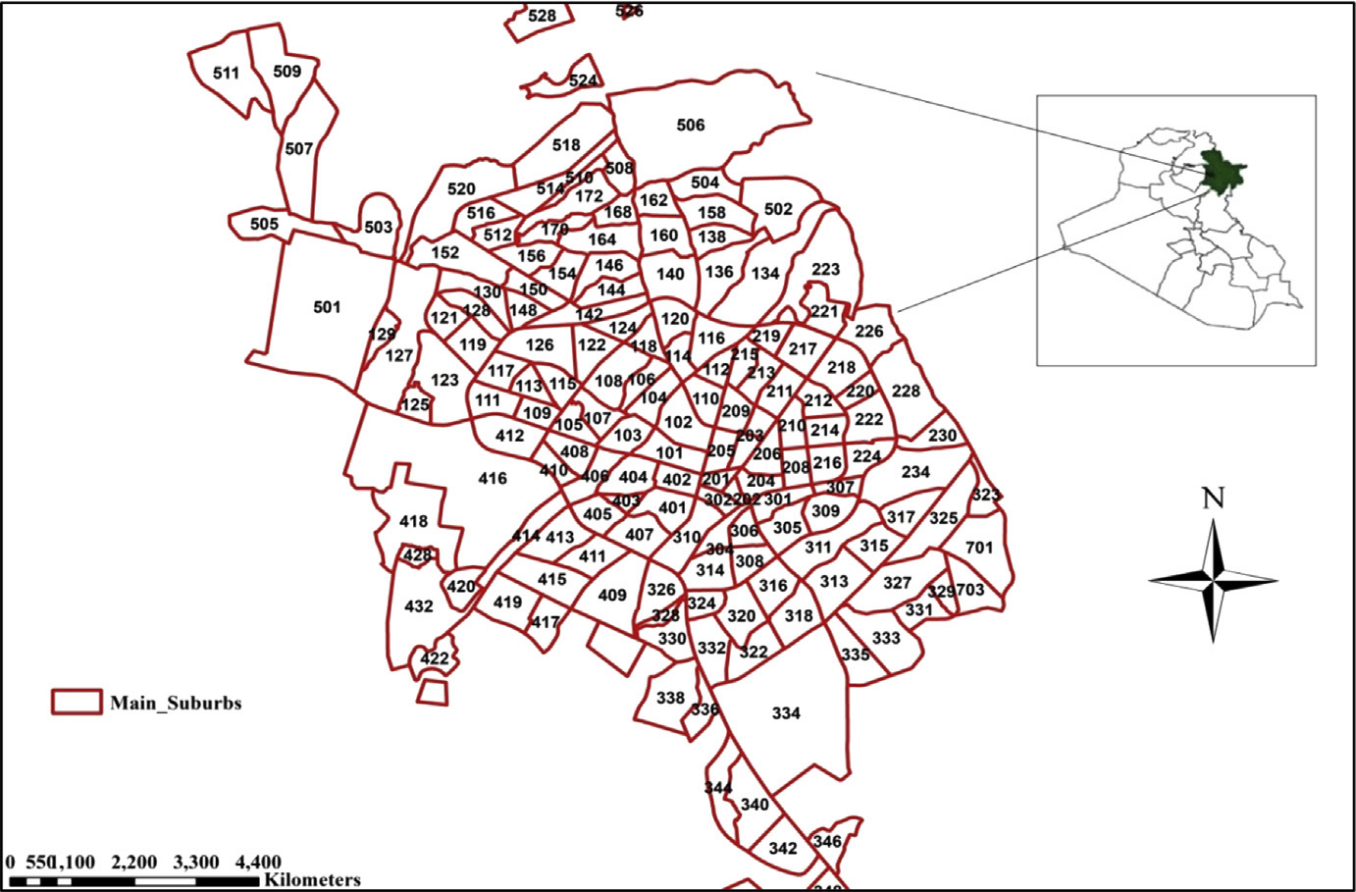
The districts of the study area (Sulaimani Municipality, 2017).

This study is carried out in Sulaimania City, Iraq - Kurdistan. The city has a mountainous topographic with elevations ranged from (645–1075 amsl). The latitudes are between (35° 36′ 07″ N - 35° 31′ 35″N), and the longitudes are between (45°22′ 23″E - 45° 28′ 23″E). Sulaimania City divided into four suburbs, which are the main suburbs, Bakrajo, Rapareen and Tasluja. The research focused on Sulaimania Main suburbs only, and the study area named as Sulaimania City. The case study total area is 114 km^2^ with 156 districts as shown in Fig. 1 (Sulaimani Municipality, 2017).

**Fig. 2:**
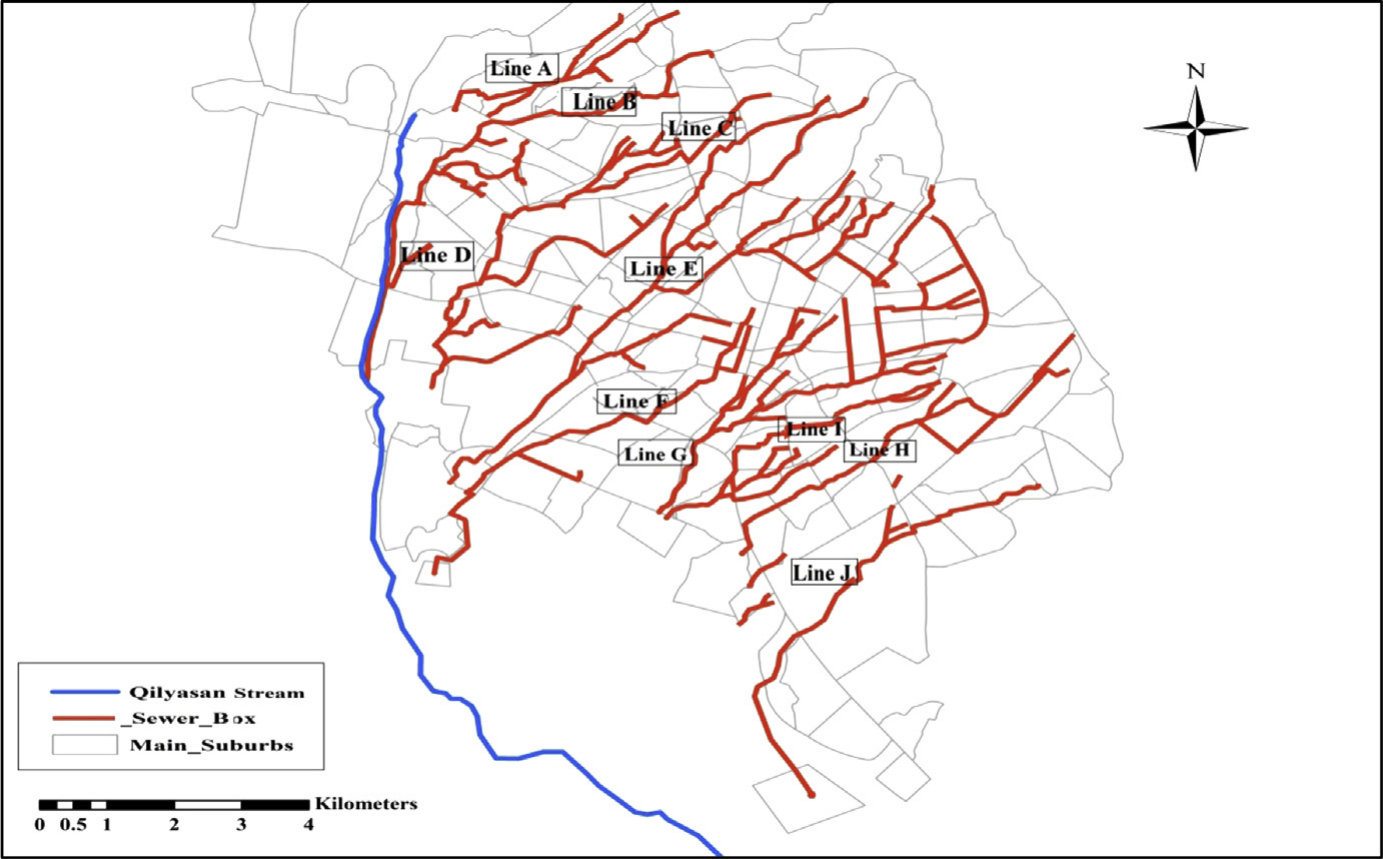
The main sewer box layout of sulaimania city (Directorate of Sewage of Sulaimani, 2017),

**Fig. 3:**
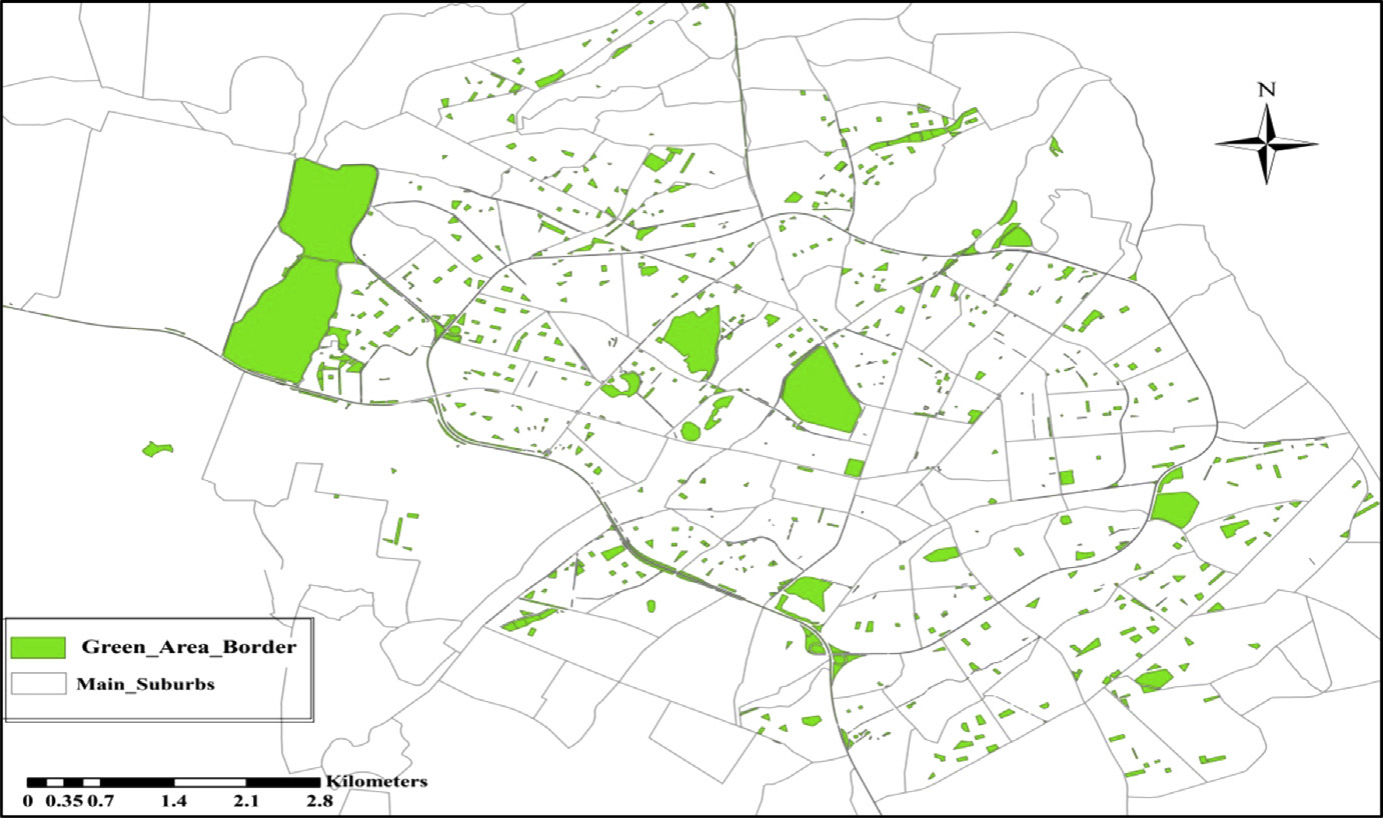
Green areas in sulaimania city (Sulaimani Municipality - Gardens DEPT., 2017).

The sewer system of the city is combined and concrete box sewers used as main trunk sewers. The arrangements of the main sewer networks consist of 10 separate groups. The groups were named as; Lines A, B, C, D, E, F, G, H, J, and I. Each sewer line is divided into branches as shown in Fig. 2. At the end of each main sewer boxes, the wastewater is currently discharged to open areas through separate outlets then to Qilyasan Stream (without any treatment). The arrangements of the sewer networks of Sulaimania City are suitable to be used in decentralized wastewater treatment systems. The city suffers from a lack of water because of the rapid expansion of the city, climate changes and immigration from surrounding areas. There are many green zones in Sulaimania City like green parks of different sizes, green zones in the road medians and the green regions inside many residential compounds. Fig. 3 shows the locations of the green areas of Sulaimania City. The total green land size is about 6.58 km^2^ (Sulaimani Municipality - Gardens DEPT., 2017), excluding the green areas inside the residential compounds.

## 3 Methodology

The study methodology and technique consisted of multiple works such as site visits to collect data and information, GIS works for mapping and modeling, Multi-Criteria Decision Model (MCDM) and statistical analysis to solve the model. The details are explained in the following sections;

### 3.1 Site visits and preliminary selection of the nominated lands

**Fig. 4:**
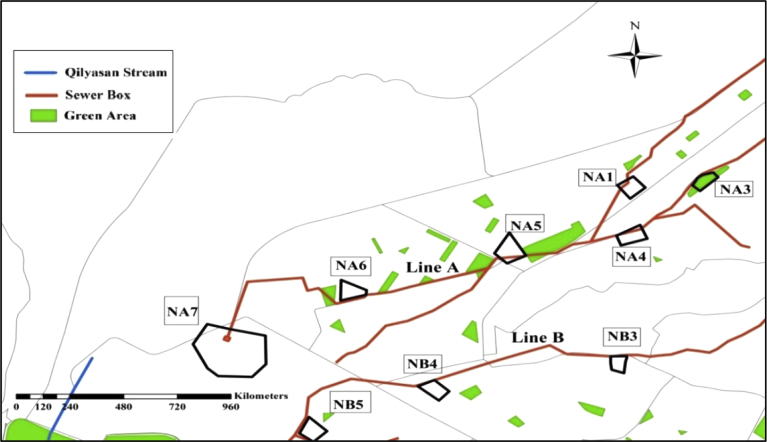
Nominated Areas on Line A and part of Line B.

Site visits and many interviews with authority representatives to collect information about the study area were done. Preliminary Selection of the Nominated Lands was done and selection was made based on many criteria, which are explained hereafter: (1) Size of the selected lands was more than 1,000 m^2^ (Gutterer et al., 2009), (2) Locations are not at the beginning of the sewer network as there will not be enough flow to treated, (3) Selected locations have accessibility to the roads, (4)Selected lands are not located on a high level area in compare to the sewer box level, and (5)Selected lands are located inside or close to the green regions. Based on the mentioned criteria preliminary selections of 134 nominated lands are obtained. The areas gathered into 10 groups which are; NA, NB, NC, ND, NE, NF, NG, NH, NI, and NJ and they are located on sewer lines A, B, C, D, E, F, G, H, I, and J respectively. Fig. 4 shows part of the selected, nominated areas on lines A and B.

### 3.2 Multi-Criteria Decision Model (MCDM)

Multi-Criteria Decision Model (MCDM) used for the suitability classifications of the proposed decentralized wastewater treatment unit's locations. Five suitability criteria used: (1) The size of the nominated lands, (2) The distances from the nominated sites to the green areas, (3) The slopes of the designated areas, (4) Population densities of the district where the decentralized units will be placed and, (5) The depth of the sewer box at the nominated area's location. Two restriction factors are used in the model which are: (1) The minimum distances of places of the nominated areas is 10 m away from the surrounding buildings (Colorado, 2017) and, (2) The maximum distance from main sewer box to the chosen areas is taken to be 50 m. After gathering the data, the information is connected and put into different layers in GIS. Weighted Linear Combination (WLC) algorithm used in the model. The suitability criteria are multiplied by the product of the area restrictions to find the land suitability index as shown in Eq. (1) used by (Urban Water Security Research Alliance, July 2012):

(1) $$\text{S}=\;\sum_{i=1}^{n}\left(Wi\;.\;Ci\right)\;\;\;\prod_{j=1}^{m}r_{j}$$

where

*S* Land Suitability Index.

*Wi* Weight of the criteria.

*C*_*i*_ Suitability of criteria.

$r_{j}$ The restrictions.

*n, m* Number of criteria and restrictions, respectively.

The five criteria are measured in different scales; therefore, they are standardized using GIS Reclassify Tool. Each criterion is weighted (Wi) based on their significance level and they are applied into Eq. (1). GIS software is not capable of finding those weights (Wi); therefore, Analytic Hierarchy Process (AHP) is used (Saaty, 1990) which is one of the Multi - Criteria Decision Making methods. Each criterion is evaluated by using pairwise comparisons matrix with scales shown in Table 1.

**Table 1:** Scale for pairwise comparisons (Saaty, 1990).

Intensity of Importance	Definition
1	Equal importance
2	Equal to moderate importance
3	Moderate importance
4	Moderate to strong importance
5	Strong importance
6	Strong to very strong importance
7	Very strong importance
8	Very to extreme importance
9	Extreme importance

#### 3.2.1 The suitability Criteria's in the AHP

In this method, the magnitude of preference between factors is reflected. The influence of the factors is specified based on experience and wise judgment as shown below:

**The area size** criterion is the preferred factor in comparison to the other factors as the land values are high inside the city. Moreover, obtaining lands inside the study area is difficult.

**Distance to the green lands** is second preferred factor as it has a significant effect on the cost of reusing the treated wastewater for irrigation. The city has a high feature, and far distances will need pumping to convey the treated wastewater.

**The slope factor** has less effect among the other suitability criteria, as it is not difficult to change the nominated area's level and make it flat. The cost of leveling the field is less than the land value and less than the cost of water conveying.

**Population density** also is essential as treatment units in crowded areas may not be accepted by the people, and it needs additional precautions and expenses. From practical experience, the additional precaution cost is still less than the value of the land and cost of the distance to the green areas.

**Depths of the sewer box** is evaluated from practical experience, and it is clear that for deep sewers pumps will be required to lift the sewage to the treatment units, which is not preferred. The costs of pumps are almost the same cost of conveying the treated wastewater to green areas but less than the value of the lands and more than the cost of the land flatting. Table 2 shows the Pairwise Comparison Matrix for the five mentioned criteria.

**Table 2:** The pair-wise comparison matrix of the 5 criteria.

Suitability Criteria	Nominated Area size	Distance to Green Area	Slope	Population Density	Depth of the Sewer Box
Nominated Area size	1	2	3	2	2
Distance to Green Area	0.5	1	2	2	1
Slope	0.33	0.5	1	0.5	0.5
Population Density	0.50	0.5	2	1	1
Depth of the Sewer Box	0.50	1	2	1	1
Sum	**2.83**	**5**	**10**	**6.5**	**5.5**

Consistency Ratio (*CR*) is calculated (Saaty, 1980) as in bellow:

$$CR=\frac{CI}{RI}\;,\;CI=\frac{\left(\lambda max-n\right)}{\left(n-1\right)}$$

Where

*n* Number of criteria.

*CI* Consistency Index.

*RI* Random Index value referred from Table 3.

**Table 3:** Random Index value RI (Saaty, 1980).

n	1	2	3	4	5	6	7	8	9	10
RI	0	0	0.58	0.9	1.12	1.24	1.32	1.41	1.45	1.49

*λmax* Largest Eigen Value.

### 3.3 The suitability criteria in the GIS

**Fig. 5:**
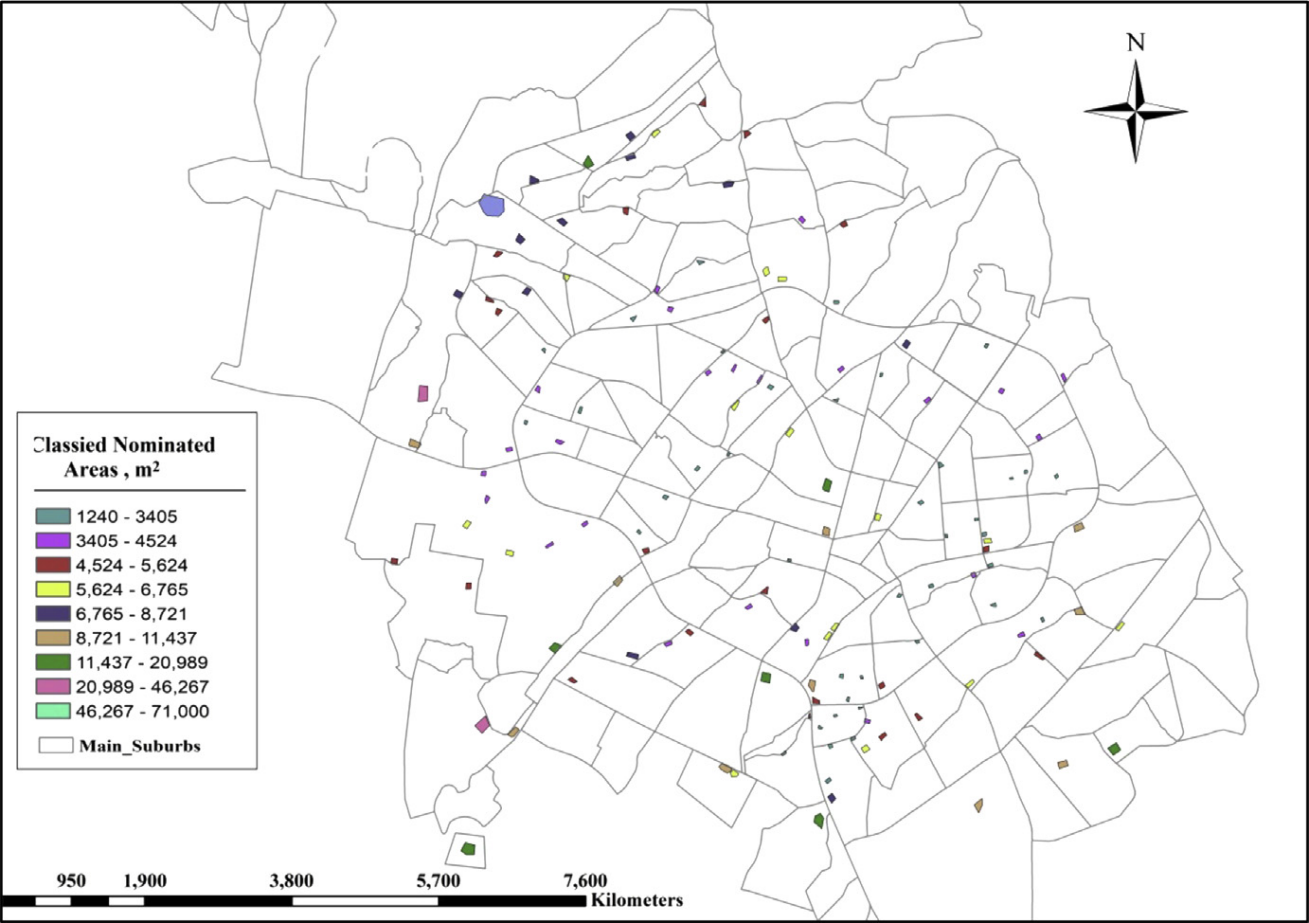
Classified nominated areas – based on size of areas.
(2) **Distance to the Green Areas**: In the GIS the distances to the green areas from the nominated lands are classified into 7 classes. The closest distance will take the most prominent rank as once the distance is close the cost of conveying water to the green areas will be less. Fig. 6 shows the classifications of the distances to the green lands.

(1) **Size of the Nominated Area**: The sizes of nominated areas classified into 9 classes in the GIS. The largest area will take the higher rank. Significant areas mean large decentralized wastewater treatment units and high flow will be reused for irrigation. Fig. 5 shows the classified nominated regions.

**Fig. 6:**
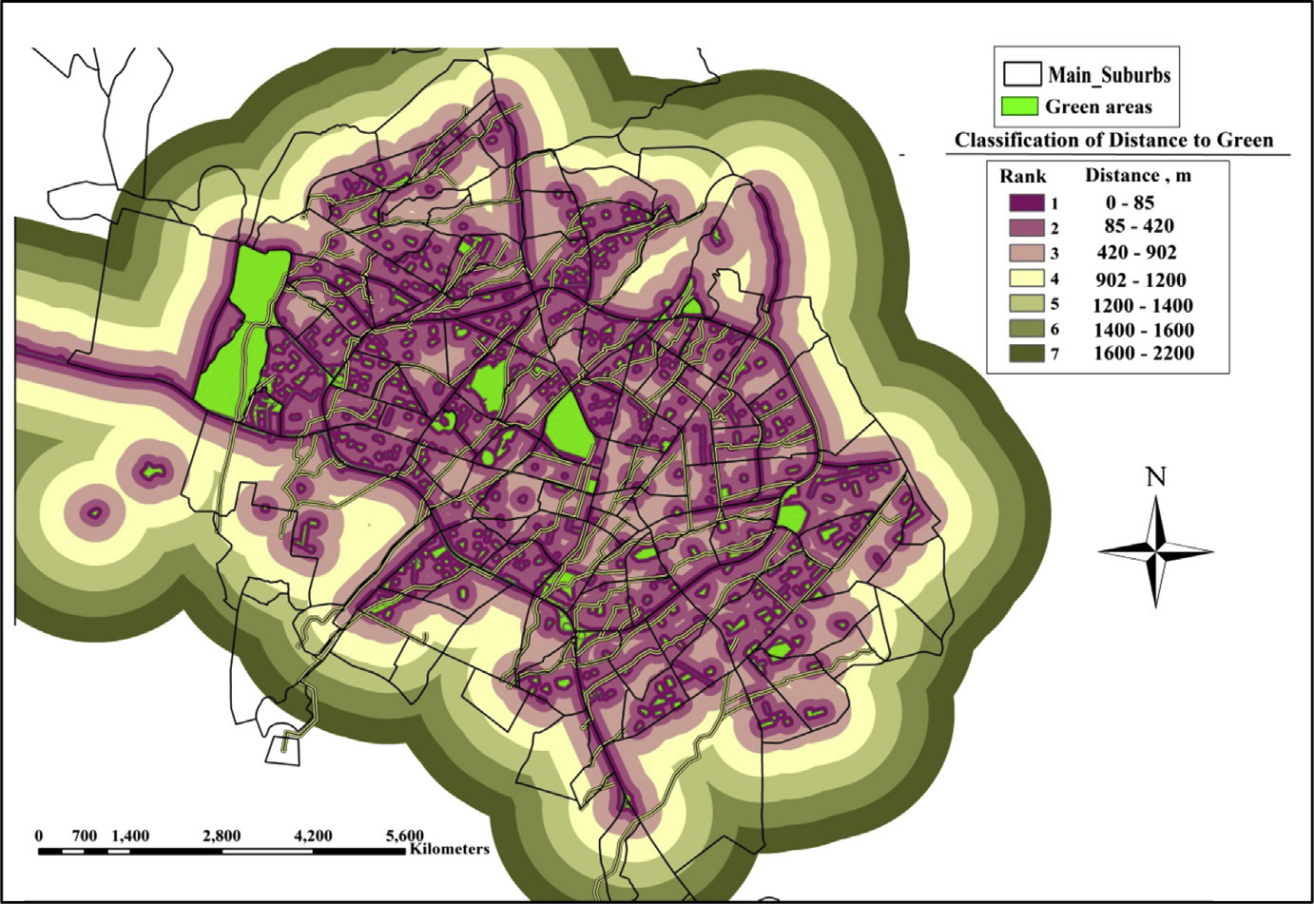
Classifications of distance to green area.
(3) **The slope of the Nominated Areas**: Flat areas are preferred and slopes of the study area (Sulaimania City) classified into 5 classes in the GIS. Fewer land slopes will take higher rank as it is more suitable for decentralized units to installing in flat lands regarding construction and operation. Fig. 7 shows the classification of the slope of the city land.

**Fig. 7:**
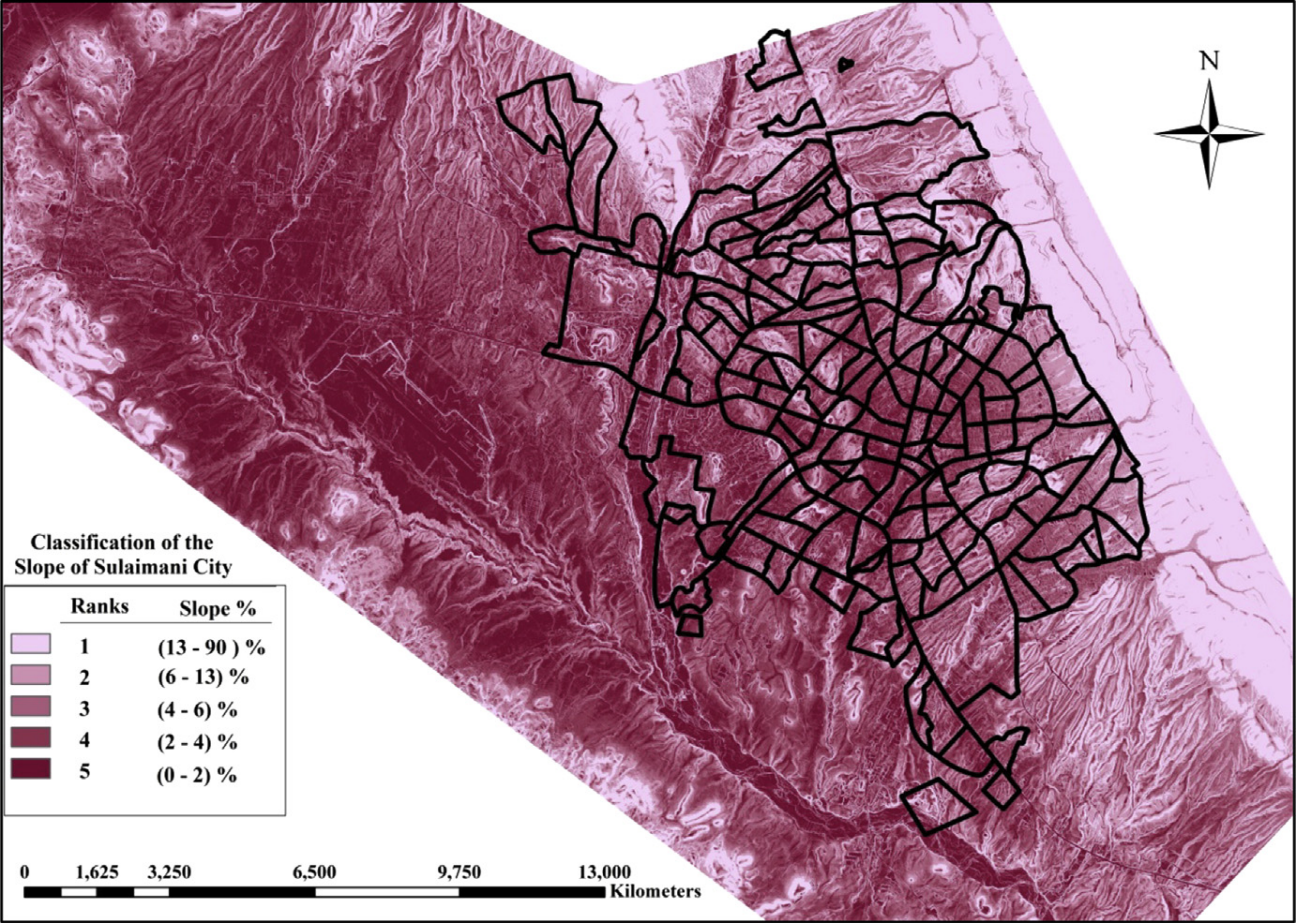
Classification of the slope of the study area.
(4) **Population Density**: Information about the population of Sulaimania City from Directorate of Statistics of Sulaimania was taken for each district of the city (Directorate of Statistics of Sulaimani, 2017). The population density of the communities where the nominated lands are selected calculated individually. There are significant differences in the population densities of districts of the study area. Old areas are crowded, and the new regions are less crowded. The population densities classified into 7classes in the GIS. Areas with low population density will take high ranks, as it is not preferred to install decentralized wastewater units in crowded areas. Fig. 8 shows the classifications of the population density of Sulaimania City.

**Fig. 8:**
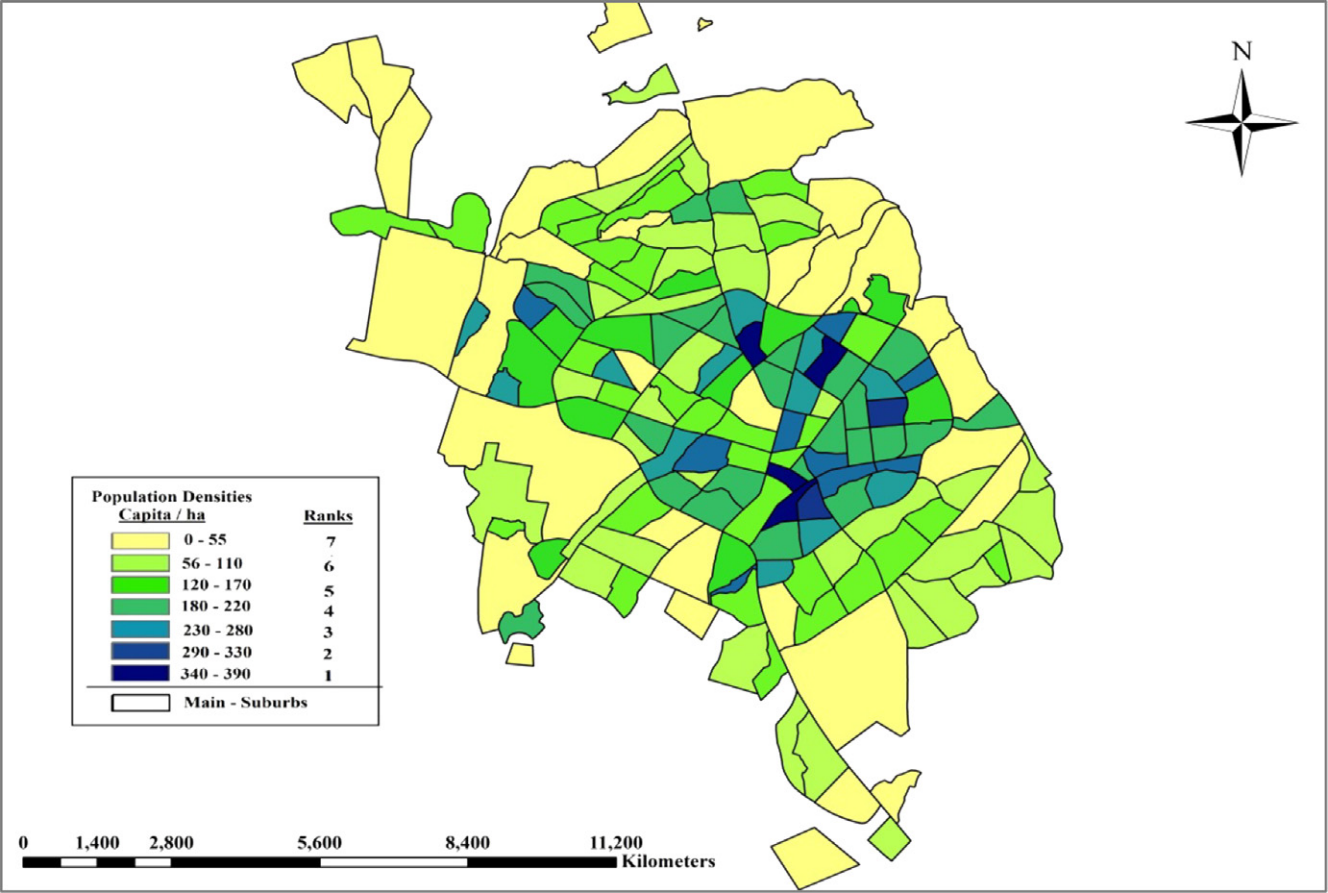
Classification of the Population Density of Sulaimania City at each Suburbs.
(5) **The depth of the Sewer Box at Nominated Areas**: The depth of the sewer box in the location of the nominated areas is essential, as it will specify the need of using pumping to lift the wastewater from deep sewer box to the treatment units. The depths calculated from the ground to the top of sewer box. The depths are ranged from 1.3 to 9.2 m and are classified into 7 classes. The higher rank will be for small depths. Fig. 9 shows the classification of the nominated areas based on the depth of the sewer box.

**Fig. 9:**
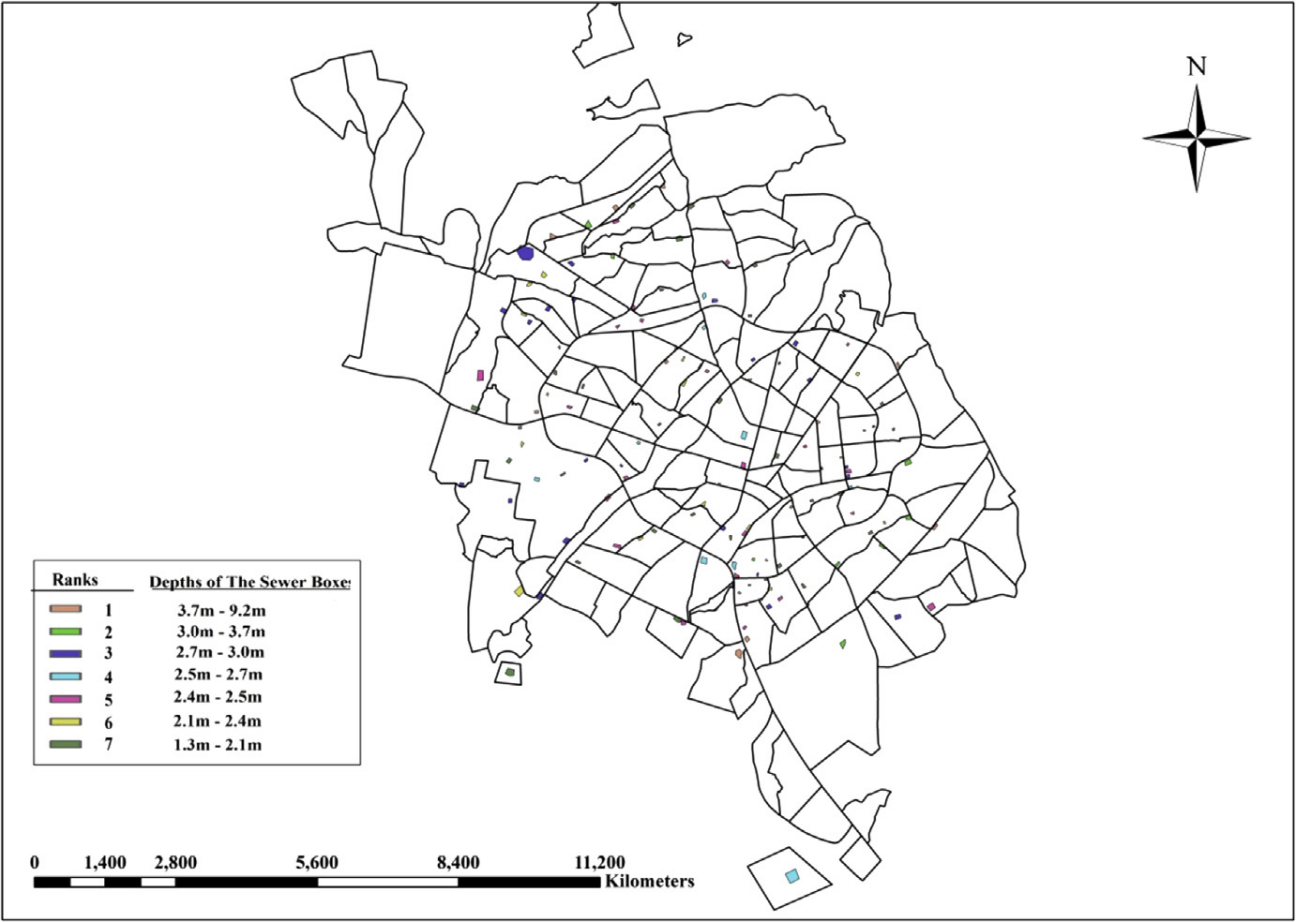
Classification of the nominated areas based on the depth of the sewer box at nominated area.

### 3.4 The restrictions

**Fig. 10:**
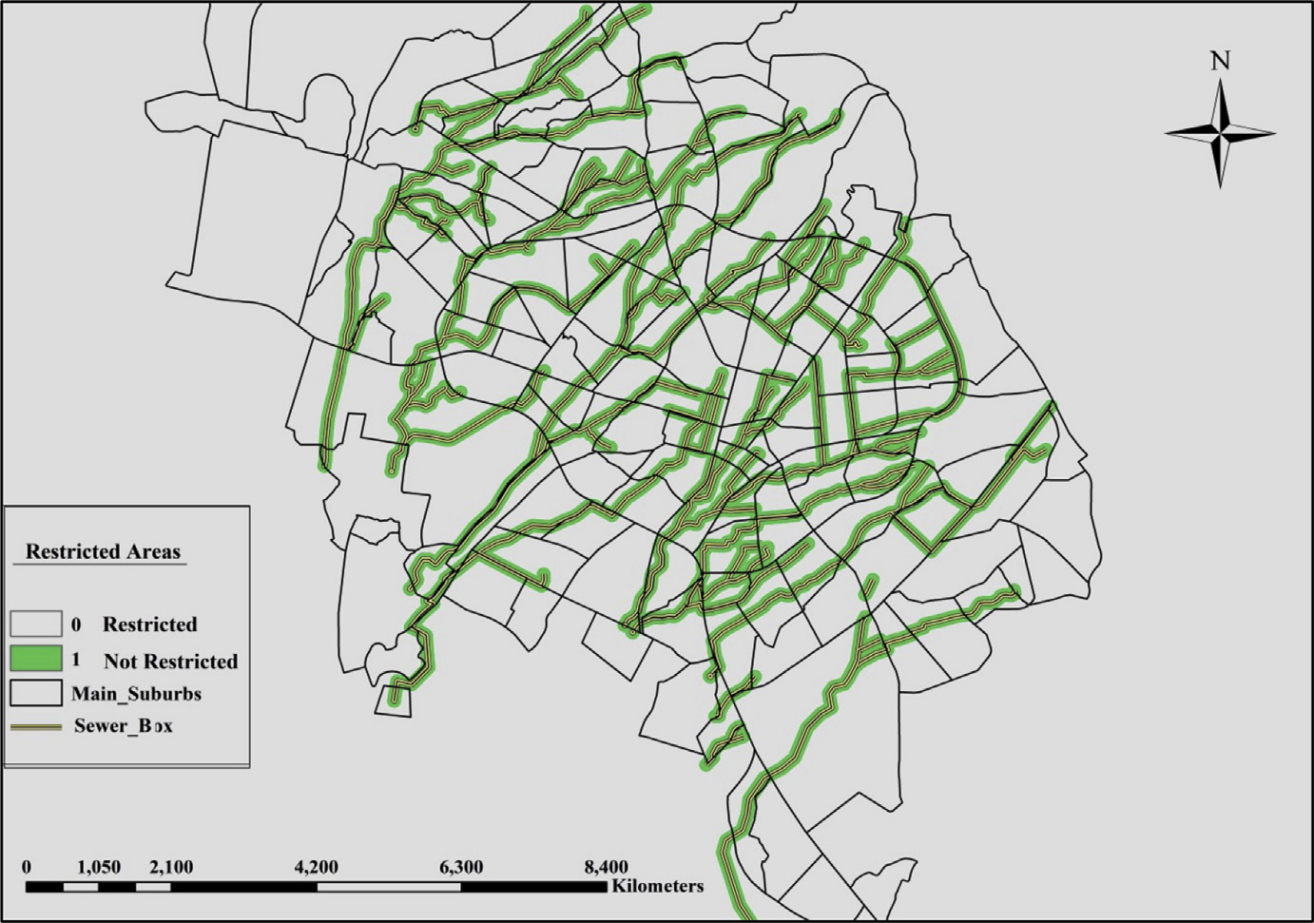
Restricted areas around the sewer box.

**Fig. 11:**
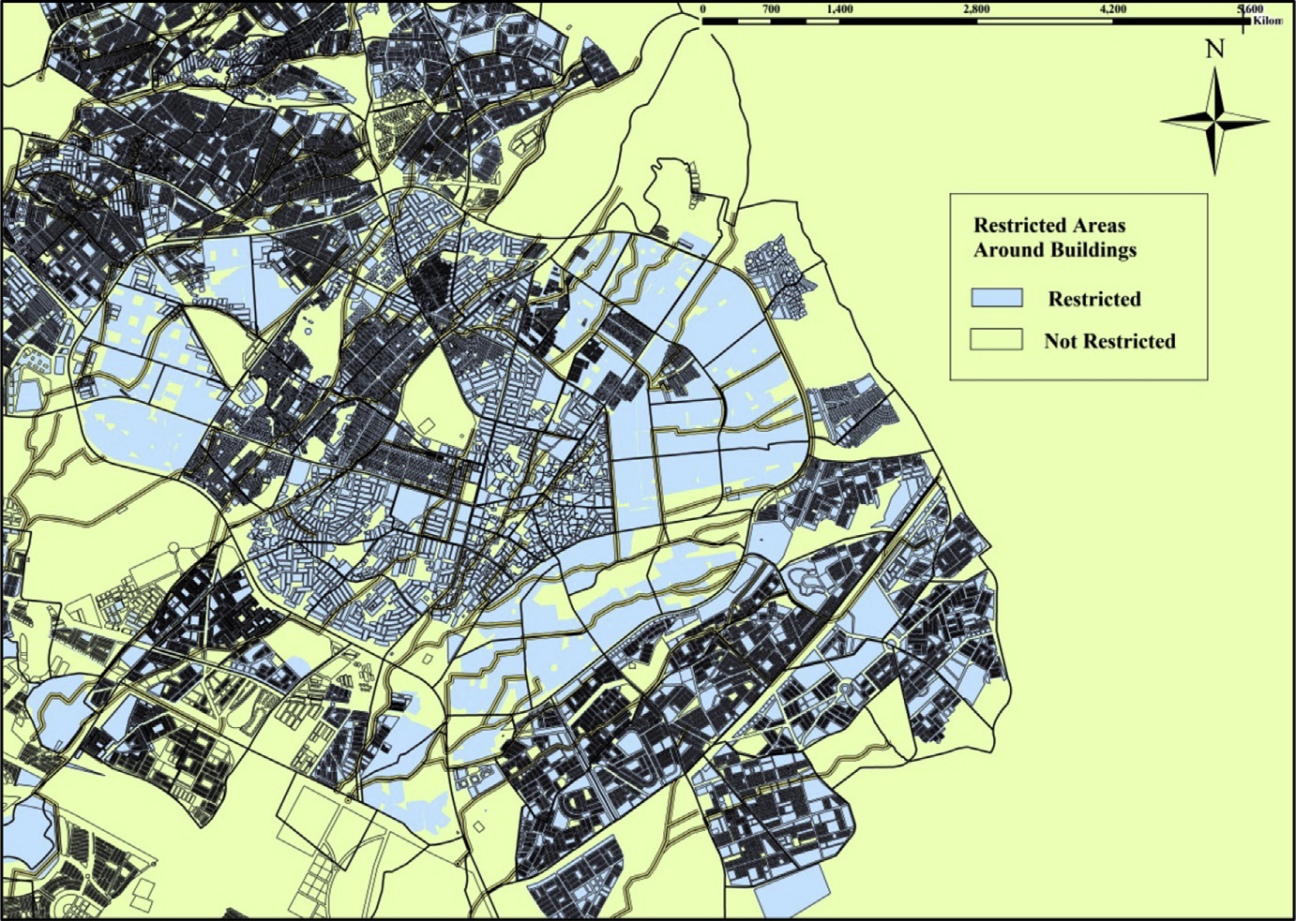
Restricted areas around the buildings.

The nominated areas should be close to the main sewer box to avoid high costs of connection works from the proposed decentralized units to the sewer box and to keep construction work as far as possible from the residential areas. That distance from the nominated areas to the residential buildings are taken based on the characteristics of the area such as average street widths and the distributions of the buildings. The width of the city's main street is 20 m, while the street widths inside residential areas ranged from (5–10) m or less in some places (Sulaimani Municipality, 2017) and the buildings arrangement are close to each other. Therefore, a distance of more than 50 m will cause a significant cost of excavation, construction, and destruction of the surrounding area. In the GIS the sewer box line is buffered with a distance of 50 m from each side. Values within the buffer area (green color) will take a Boolean value of one while values outside the buffer area are the restricted area, and it will take a Boolean value equal to zero. Fig. 10 shows the restricted area around the sewer box. According to the environmental restrictions, the proposed decentralized unit should be far away from the residential buildings at least by a distance of 10 m (Colorado, 2017). Therefore; the building layer is buffered with a range of 10 m in the GIS model. The restricted areas are inside the buffer region and it has a Boolean value of zero (light blue color). The area outside the buffered area is the allowable areas, and it has a Boolean value equal to one. Fig. 11 shows the details of the buffered areas around the buildings.

### 3.5 Structure of the GIS modeling

**Fig. 12:**
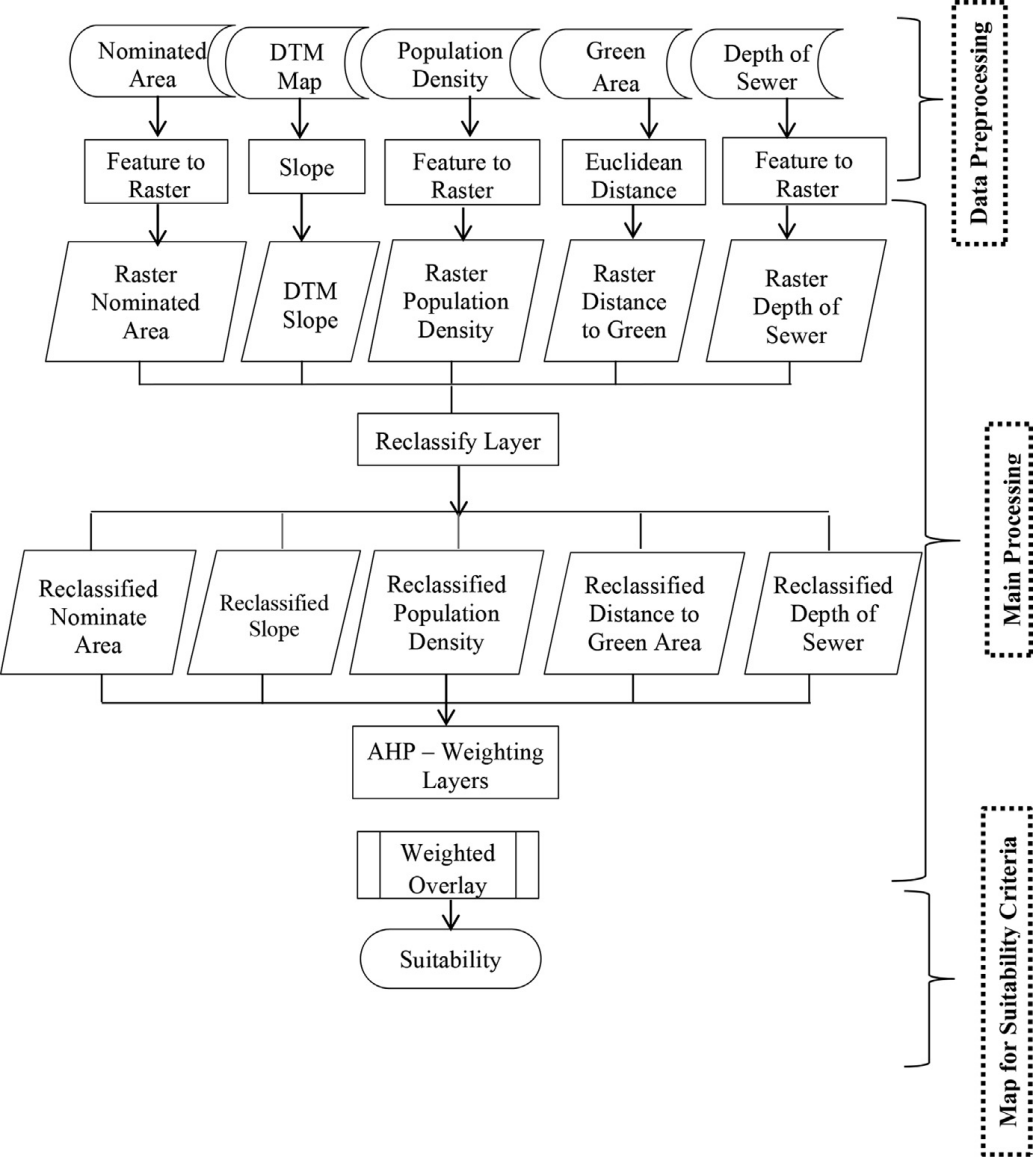
Flow chart of GIS suitability model structure.

**Fig. 13:**
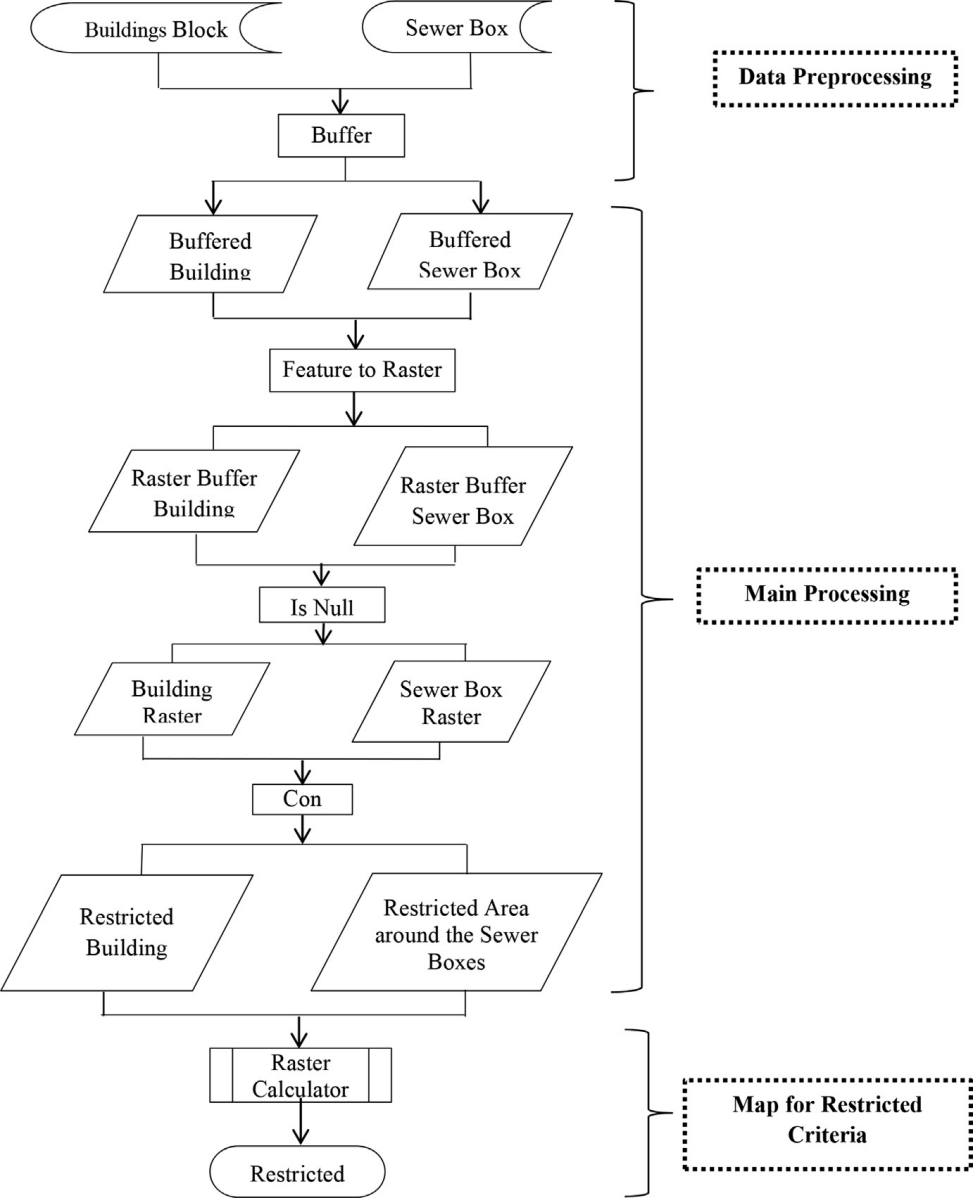
Flow chart of GIS restriction Model's structure.

**Fig. 14:**
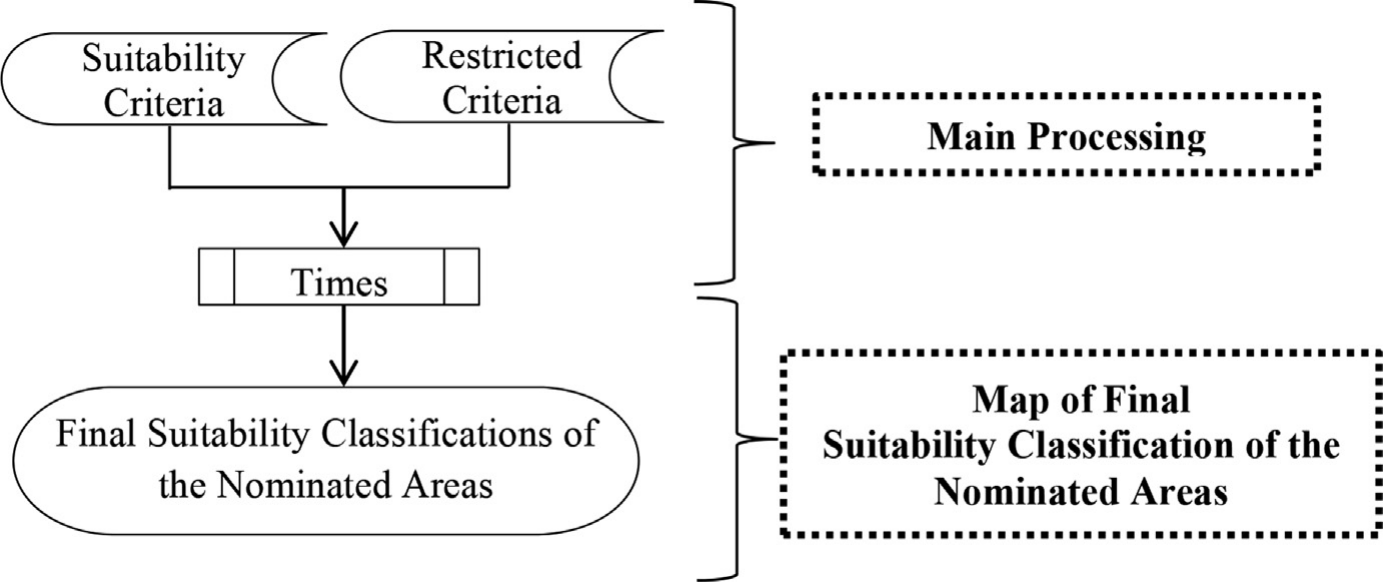
Flow Chart of GIS Suitability Classifications of the Nominated areas.

In this research, Eq. (1) applied into ArcGIS software by creating three GIS models: (1) Suitability Model, (2) Restriction Model and the (3) Suitability Classifications of the Nominated areas. Figs. 12, 13, and 14 illustrate the flowcharts of the structure of the three models respectively. The conceptual approach of the application of those three models into GIS is based on three main steps: data input and pre-processing, primary processing, and output maps identifying the locations' suitability. In the Suitability Model, the values of $\sum_{\text{i}=1}^{\text{n}}\left(\text{Wi}\;.\;\text{Ci}\right)$calculated, and the weights of each criterion measured from the AHP method applied in the Weighted Overlay Tool. The results of running the suitability model are 7 suitability classes. The Restriction Model is used to calculate the product of the area restrictions $\prod_{\text{j}=1}^{\text{m}}{\text{r}}_{j}$. The third model performed by multiplying the Suitability Model times the Restriction Model and 6 classes of suitable nominated areas obtained as shown in Table 4. Each nominated area divided into parts that have a specific suitability class. For instances if a nominated area has an area of 1000 m^2^ and that area has a restricted part equal to R = 100 m^2^, Suitable part equal to S = 250 m^2^ and Highly Suitable part equal to H.S. = 650 m^2^, that means the remaining classes are zero in this nominated area. Another nominated area has different class values.

**Table 4:** Suitability Classifications of the Nominated Areas, m.^2^.

Restricted (R)
Moderately Suitable (M.S.)
Suitable (S)
Very Suitable (V.S.)
Highly Suitable (H.S.)
Extremely Suitable (E.S.)

### 3.6 Weighted average value (WAV)

The results that obtained from the AHP and GIS models are only to classify the suitability of each of the 134 nominated areas. It is required to make a further selection from those areas to find the optimum locations of the treatment units. Normalized Weighted Average Value (NWAV) of the final GIS model's results of each area calculated as shown below:

The weighted average value (WAV) of each nominated area is found by applying Eq. (2):

(2) $$\text{WAV}\;=\;\frac{\left(\text{R}\;\text{x}\;0.0\right)\;+\;\left(\text{M}.\text{S}\;\text{x}\;0.2\right)\;+\;\left(\text{S}\;\text{x}\;0.4\right)\;+\;\left(\text{V}.\text{S}\;\text{x}\;0.6\right)\;+\;\left(\text{H}.\text{S}\;\text{x}\;0.8\right)\;+\;\left(\text{E}.\text{S}\;\text{x}\;1.0\right)}{3}$$

In Eq. (2), each nominated area's suitability classes values (in m^2^) multiplied by weight ranged from 0.0 to 1.0. Those weights specified according to the preference of each class. For instance, R is restricted; therefore it's weight is equal to 0.0 while the weight of E.S class is equal to 1.0. The amount of WAV of each nominated area is normalized to make it easier when selecting the best suitable location as shown in Eq. (3):

(3) $$Normalized\;WAV=\;NWAV=\;\;\frac{\left(WAV-\;min\right)}{\left(max-\;min\right)}$$

where:

min, max; Minimum and Maximum value of WAV of nominated areas located on each sewer box, The values of NWAV are ranged from 0.0 to 1.0.

The selection of the final best locations is based on the NWAV's amount. For the 10 nominated areas group, sites that have the highest values of NWAV are selected.

#### 3.6.1 The treatment plant type

Extended aeration package plants are used as it is recommended for small residential communities. Extended aeration method is a modified activated sludge process used to remove biodegradable organic wastes under aerobic condition. This type of plant is recommended, as it is efficient, does not need big footprints and it produces a small amount of sludge (Metcalf & Eddy, INC., 1991).

## 4 Results and discussion

In the present study, the results of the weights of the suitable criteria using (AHP) method shows that the weight W of the size of the nominated area's factor has the most considerable effect which is equal to 35 % and the other results are shown in Table 5.

**Table 5:** The weight (W) of the 5 criteria.

Suitability Criteria	Weigh (W), in %
The size of the nominated area	35
Distance to the green areas	21
Slope	10
Population density	16
Depth of the sewer box	18

**Fig. 15:**
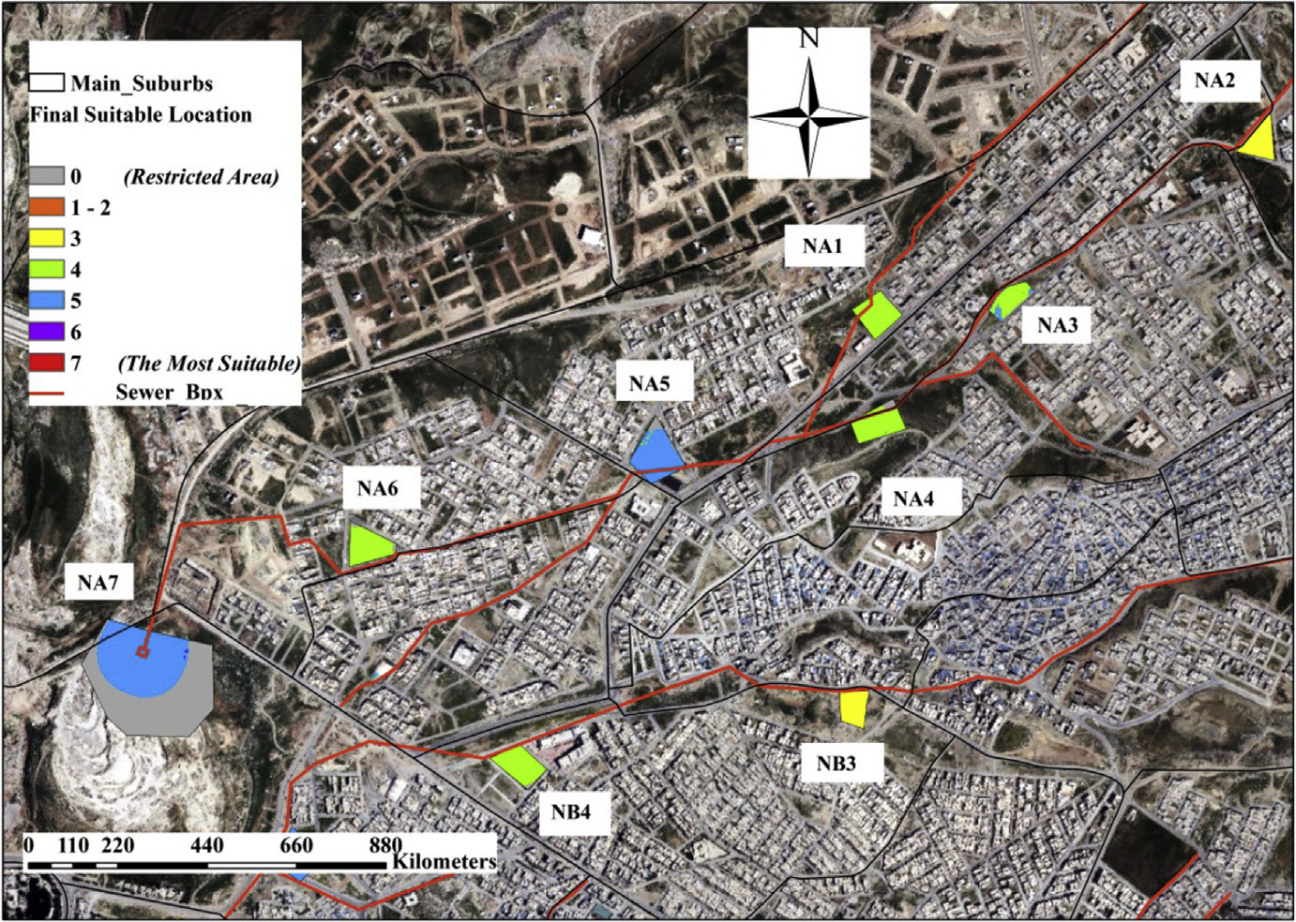
Suitability results of nominated areas NA1, NA2, NA3, NA4, NA5, NA6, NA7, NB3 and NB4.

The Consistency Ratio (CR) is found to be equal to 1.63% < 10 %, which is acceptable, and it means that the judgment of criterion's ranking was correct. The results of the GIS model showed the percentages of the suitability classification of the total nominated areas as presented in Table 6. The results also showed that most of the nominated areas could not be classified under a certain suitability class with 100% ratio. For example, nominated area NE18 has 3.4% restricted area (R = 91 m^2^), 1.5 % is suitable (S = 40 m^2^), and 95.1% is very suitable. (V.S. = 2550 m^2^). Fig. 15 shows the suitability of nominated areas NA1, NA2, NA3, NA4, NA5, NA6, NA7, NB3, and NB4.

**Table 6:** The percentages of the suitability of the nominated areas.

The classifications	Area, m^2^	Percentage % of Total Area
Restricted (R)	69456.76	8.5
Moderately Suitable (M.S.)	2862.949	0.4
Suitable (S)	104679.1	12.8
Very Suitable (V.S.)	316335.7	38.8
Highly Suitable (H.S.)	263310.7	32.3
Extremely Suitable (E.S.)	59184.42	7.2
**Total Area of the Nominated Areas of the City**	**815,829.7**	**100 %**

The analysis showed that 58 nominated areas have some restricted parts. Most of those restricted parts are located at the outer part of the nominated land, and others located along one side of the area, or at one corner of the land. The restricted % part in the nominated areas are; 20 areas have (5–15) %, 31 areas have <5 %, 6 areas have >15 %, and the remaining areas have no restricted part. The size of the other restricted parts of the remaining 43 nominated areas varied from 393 m^2^ to 10 m^2^ and the restricted locations are mostly at the outside part of the lands. The other nominated areas have percentages of suitability ranged from a suitable class to extremely suitable. The results also showed that (16) nominated location have suitability classification ranged from very suitable to highly suitable with a percentage >75%. In addition, it is found that only 3 areas are extremely suitable with a percentage >90%.

From the results of the final suitability of the GIS for each nominated area (134 area), NWAV is calculated. The values of NWAV reflect the level of suitability of the location to be used for installing decentralized treatment unit. For instance, nominated area NB7, the total area is 5544 m^2^, which has 262 m^2^ restricted (R), 161 m^2^ suitable (S) , 1521 m^2^ very suitable (V.S) and has no other classification levels (M.S = 0, H.S = 0 and E.S. = 0). R % = (262/5544) x 100 = 4.7 %, M.S % = (0.0/5544) x 100 = 0.0 %, S% = (161/5544) x 100 = 2.9 %, V.S%. = (1521/5544) x 100 = 92%, H.S.% = (0.0/5544) x 100 = 0.0 %, and E.S.% = (0.0/5544) x 100 = 0.0 %.

WAV = [(4.7 × 0.0) + (0.0 × 0.2) + (2.9 × 0.4) + (92 × 0.6) + (0.0 × 0.8) + (0.0 × 1.0)]/3.

WAV = 13.

NWAV = (13–7)/(26–7) = 0.3,

[The minimum value of WAV of line B = 7 and the maximum value of WAV of line B is 26].

**Fig. 16:**
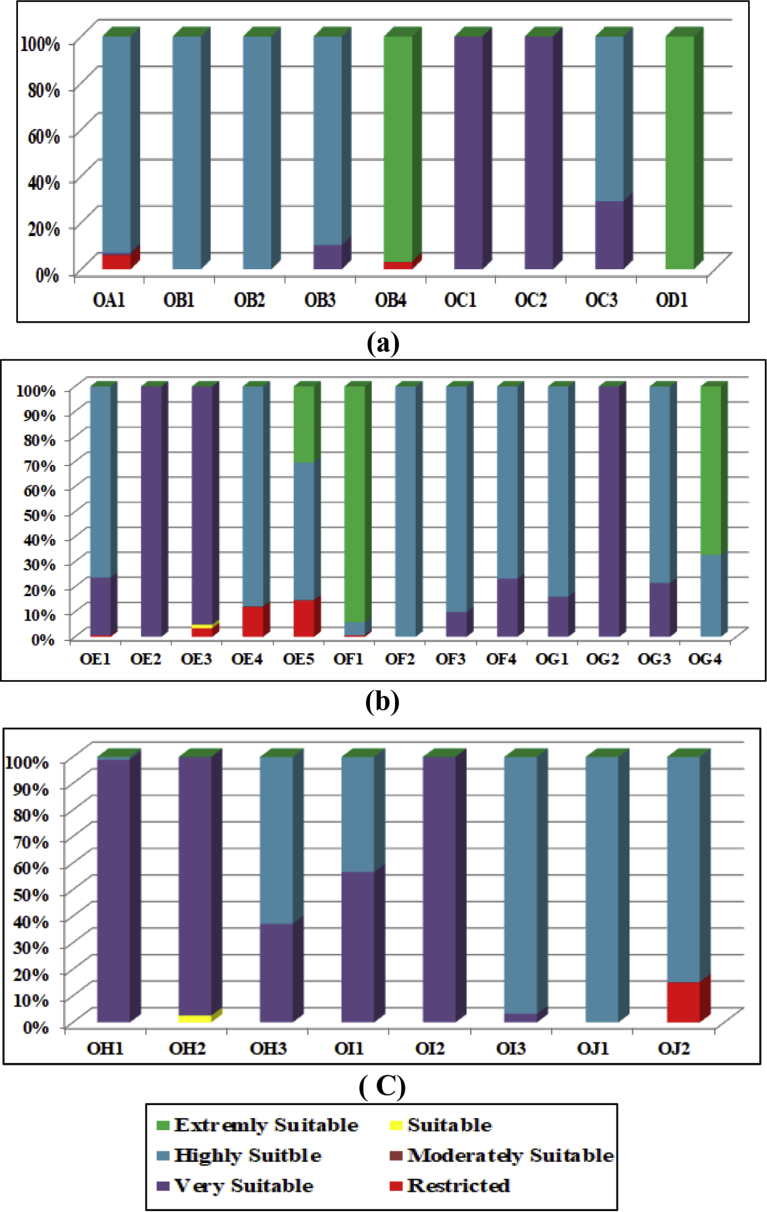
The Suitability Classifications of the Optimized Nominated Areas on lines: (a) on Lines A, B, C and D, (b) on Lines E, F, and G, (c) on Lines H, I and J.

The values are ranged from 0.0 to 1.0 with an average amount equal to 0.50. The optimum locations from the 134 nominated areas are the areas that have the highest NWAV. From each group (10 groups) some nominated regions are selected, who their NWAV ≥0.50. The total selected areas are 30 optimum locations as shown in Table 7. Figs. (16a, 16b and 16c) show the suitability classifications of the 30 optimum nominated areas.

**Table 7:** The selected optimum 30 nominated areas and the NWAV of each area.

Nominated Area	Line	NWAV	Nominated Area	Line	NWAV
OA1	A	1.0	OF2	F	0.6
OB1	B	0.7	OF3		0.5
OB2		0.7	OF4		0.5
OB3		0.6	OG1	G	0.7
OB4		1.0	OG2		0.5
OC1	C	0.6	OG3		0.7
OC2		0.6	OG4		1.0
OC3		0.9	OH1	H	0.5
OD1	D	1.0	OH2		0.7
OE1	E	1.0	OH3		1.0
OE2		0.6	OI1	I	0.8
OE3		0.6	OI2		0.6
OE4		0.9	OI3		1.0
OE5		1.0	OJ1	J	1.0
OF1	F	1.0	OJ2		0.5

**Fig. 17:**
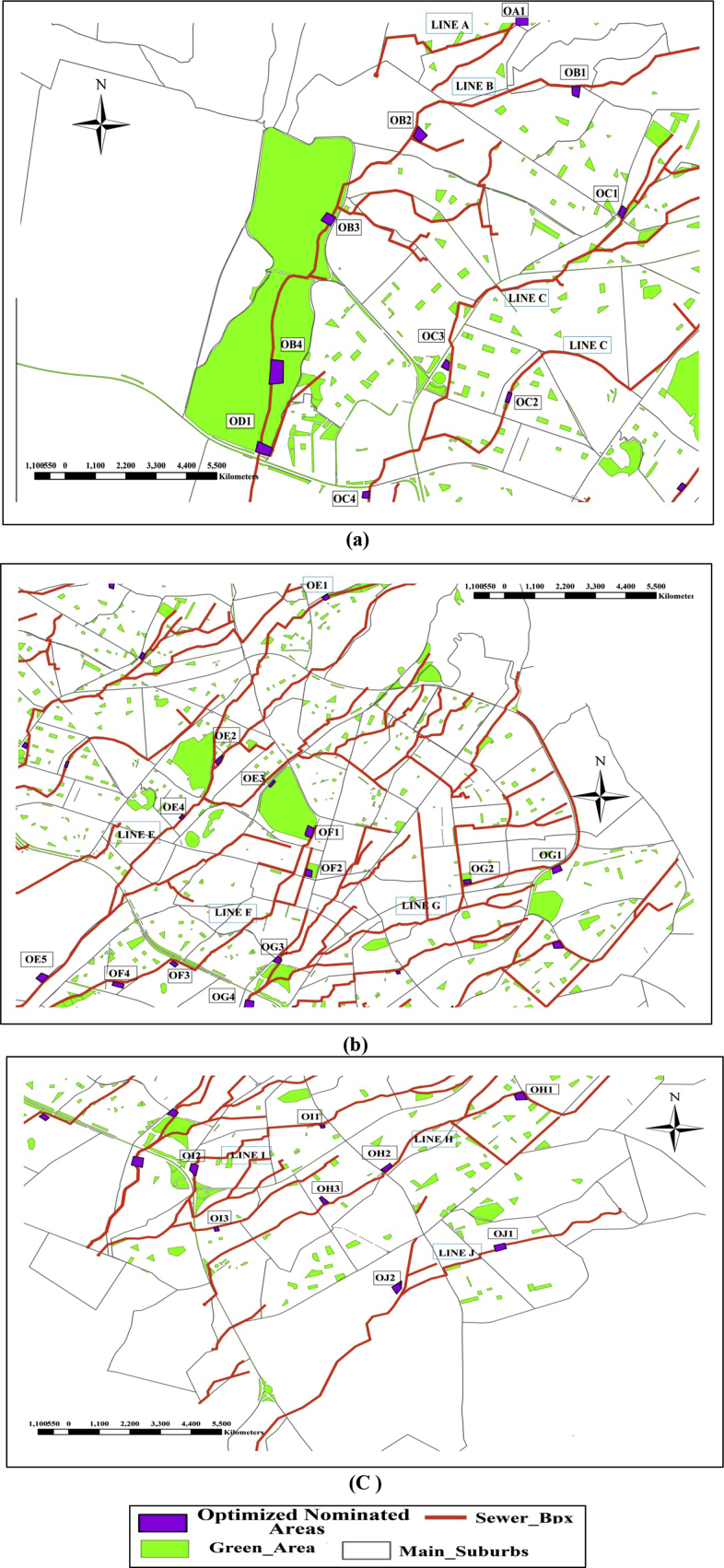
The final optimized suitable nominated areas: (a) on Lines A, b, C and D, (b) on Lines E, F, and G, (c) on Lines H, I and J.

The final 30 optimum locations are distributed in organized and strategical positions in the study area and are spread over the 10 main sewer box lines. The number of the selected regions per each sewer box ranged from 1 – 5. Line A has only one suitable area, as the preliminary areas chosen from the beginning are only 7 areas because line A is short and covers a small part of the city's districts. Figs. 17a (17-b) and (17- c) shows the 30 optimum locations of the proposed decentralized treatment units.

## 5 Conclusion

Decentralized wastewater treatment units are an effective solution to the problem of water shortage in a city like Sulaimania. Selecting the locations of the units is crucial and should be done according to the required standards. This study was conducted to find suitable sites for decentralized wastewater treatment units in Sulaimania City by using GIS and Analytical Hierarchy Process (AHP). Five suitability criteria were taken; area size, distance to green areas, population density, land slope and depth of the sewer pipes at the nominated locations. Also, two restrictions were used; distance from the decentralized unit to buildings ≥10 m and the distance from the decentralized units to the sewer box ≤50 m. Selections of 134 locations were made in Sulaimania City to test their suitability. Previous studies related to the same aspect were to find the best locations of a certain facility directly using GIS. In this research the work was done in two stages. First an evaluation of preliminary selected locations of the DTUs was done. Second stage was to evaluate the preselected locations then select the best areas. The results of the model classified the selected areas into 6 suitable classes starting from restricted to extremely suitable. Moreover, from the suitability results of GIS and AHP further analyses has been done and 30 final optimum locations found which are beside the 10 sewer lines.

## Declarations

### Author contribution statement

Ako Rashed Hama, Zeren Jamal Ghafoor: Conceived and designed the experiments; Performed the experiments; Analyzed and interpreted the data; Contributed reagents, materials, analysis tools or data; Wrote the paper.

Rafea Hashim Al-Suhili: Conceived and designed the experiments; Performed the experiments; Analyzed and interpreted the data.

### Funding statement

This research did not receive any specific grant from funding agencies in the public, commercial, or not-for-profit sectors.

### Competing interest statement

The authors declare no conflict of interest.

### Additional information

No additional information is available for this paper.
